# Supplementary material for: Organizing structural principles of the IL-17 ligand–receptor axis
Source: Nature. 2022 Jul 21;609(7927):622–9. doi: 10.1038/s41586-022-05116-y (PMC9477748; doi:10.1038/s41586-022-05116-y)
Supplement: Supplementary file 1 — Reporting Summary [file 41586_2022_5116_MOESM1_ESM.pdf]

nature portfolio

Corresponding author(s): K. Christopher Garcia

Last updated by author(s): Jul 1, 2022

Reporting Summary

Nature Portfolio wishes to improve the reproducibility of the work that we publish. This form provides structure for consistency and transparency in reporting. For further information on Nature Portfolio policies, see our Editorial Policies and the Editorial Policy Checklist.

Statistics

For all statistical analyses, confirm that the following items are present in the figure legend, table legend, main text, or Methods section.

n/a	Confirmed	
☐	☒	The exact sample size (*n*) for each experimental group/condition, given as a discrete number and unit of measurement
☐	☒	A statement on whether measurements were taken from distinct samples or whether the same sample was measured repeatedly
☐	☒	The statistical test(s) used AND whether they are one- or two-sided<br>*Only common tests should be described solely by name; describe more complex techniques in the Methods section.*
☒	☐	A description of all covariates tested
☐	☒	A description of any assumptions or corrections, such as tests of normality and adjustment for multiple comparisons
☐	☒	A full description of the statistical parameters including central tendency (e.g. means) or other basic estimates (e.g. regression coefficient) AND variation (e.g. standard deviation) or associated estimates of uncertainty (e.g. confidence intervals)
☐	☒	For null hypothesis testing, the test statistic (e.g. *F*, *t*, *r*) with confidence intervals, effect sizes, degrees of freedom and *P* value noted<br>*Give P values as exact values whenever suitable.*
☒	☐	For Bayesian analysis, information on the choice of priors and Markov chain Monte Carlo settings
☒	☐	For hierarchical and complex designs, identification of the appropriate level for tests and full reporting of outcomes
☒	☐	Estimates of effect sizes (e.g. Cohen's *d*, Pearson's *r*), indicating how they were calculated

Our web collection on statistics for biologists contains articles on many of the points above.

Software and code

Policy information about availability of computer code

Data collection	Single-molecule imaging: a Customized TIRF microscope (IX71 Olympus with EMCCD iXon DU897D, Andor Technology) was used.<br>CryoEM: data were collected on a Titan Krios (ThermoFisher) using SerialEM v3.9.0 at Stanford cEMc and SerialEM v4.0.0beta3 at Janelia Research Campus.<br>Signaling assays: HEK-Blue reporter assays and ELISA assays were read on a SpectraMax Paradigm plate reader (Molecular Devices) using SoftMax Pro v7.1.<br>Surface plasmon resonance data were collected using Biacore T100 control software version 2.0.4.
Data analysis	Single-molecule imaging: SLIMfast4C single molecule evaluation software was used and test data sets can be accessed here: https://doi.org/10.5281/zenodo.5712332.<br>CryoEM: cryoSPARC v3.1.0 and v3.2.0 were used for data analysis.<br>Surface plasmon resonance: Biacore T100 Evaluation Software v2.0.4.<br>Structural analysis: Coot v0.9.6; Phenix v1.20.1; UCSF Chimera v1.15; UCSF ChimeraX v1.3; PyMol v2.2.3; qtPISA v2.1.0; ISOLDE v1.3.0; DeepEMhancer version 20210511.<br>Signaling assay analysis and statistical analysis: GraphPad Prism v9.3.1.

For manuscripts utilizing custom algorithms or software that are central to the research but not yet described in published literature, software must be made available to editors and reviewers. We strongly encourage code deposition in a community repository (e.g. GitHub). See the Nature Portfolio guidelines for submitting code & software for further information.

Data

Policy information about availability of data

All manuscripts must include a data availability statement. This statement should provide the following information, where applicable:

- Accession codes, unique identifiers, or web links for publicly available datasets
- A description of any restrictions on data availability
- For clinical datasets or third party data, please ensure that the statement adheres to our policy

All single molecule imaging data have been deposited at Zenodo: raw images are at DOI:10.5281/zenodo.6783369 and calibration images are at 10.5281/zenodo.6787325. The CryoEM maps and atomic coordinates for the IL-17RB-IL-25 (2:2), IL-17RB-IL-25 (6:6), IL-17RB-IL-25—-IL-17RA (2:2:2), IL-17RA-IL-17A (2:2), and IL-17RA-IL-17A-IL-17RC (2:2:1) structures have been deposited in the EMDB (EMD-26833, EMD-26834, EMD-26835, EMD-26836, EMD-26837) and PDB (7UWJ, 7UWK, 7UWL, 7UWM, 7UWN), respectively. All other data are available from the corresponding author upon reasonable request.

Field-specific reporting

Please select the one below that is the best fit for your research. If you are not sure, read the appropriate sections before making your selection.

☒ Life sciences ☐ Behavioural & social sciences ☐ Ecological, evolutionary & environmental sciences

For a reference copy of the document with all sections, see nature.com/documents/nr-reporting-summary-flat.pdf

Life sciences study design

All studies must disclose on these points even when the disclosure is negative.

Sample size	Single-molecule experiments: sample sizes were estimated based on extensive statistical analysis of data obtained in a systematic assessment of the single molecule cotracking analyses (Sotolongo Bellon et al., Cell Rep Meth 2, 2022, 100165, DOI:10.1016/j.crmeth.2022.100165). Signaling assays and surface plasmon resonance: no sample size calculations were performed. For signaling and surface plasmon resonance experiments, samples sizes where determined based on previous experience with similar experiments and the sample sizes were adequate based on clear distributions in the data and clear differences among the various conditions.
Data exclusions	No data were excluded.
Replication	All attempts at replication were successful. Most results were replicated in at least two independent experiments. For some of the single-molecule and SPR conditions only one experiment was performed. The signaling experiments characterizing the IL-17RA KO and IL-17RB KO cell lines were conducted once. The number of independent biological replicates and sample sizes for all data are indicated in the figure legends.
Randomization	PBMCs were obtained from multiple donors at random. For in vitro experiments and protein structure determination, independent variables are tightly controlled and therefore covariates are not relevant to those studies.
Blinding	Blinding of investigators was not performed or necessary as the readouts of the protein structure determination and in vitro assays are not subjective to investigator bias.

Reporting for specific materials, systems and methods

We require information from authors about some types of materials, experimental systems and methods used in many studies. Here, indicate whether each material, system or method listed is relevant to your study. If you are not sure if a list item applies to your research, read the appropriate section before selecting a response.

Materials & experimental systems

n/a	Involved in the study	
☐	☒	Antibodies
☐	☒	Eukaryotic cell lines
☒	☐	Palaeontology and archaeology
☒	☐	Animals and other organisms
☐	☒	Human research participants
☒	☐	Clinical data
☒	☐	Dual use research of concern

Methods

n/a	Involved in the study	
☒	☐	ChIP-seq
☒	☐	Flow cytometry
☒	☐	MRI-based neuroimaging

Antibodies

Antibodies used	Nanobodies: Anti-GFP and anti-ALFAtag (produced in-house).
Validation	Nanobodies: Anti-GFP and anti-ALFAtag were previously validated (doi.org/10.1016/j.crmeth.2022.100165 and doi.org/10.1038/s41467-019-12301-7, respectively). Interaction of the in-house produced nanobodies with their target proteins was confirmed by quantitative binding assays.

Eukaryotic cell lines

Policy information about cell lines

Cell line source(s)	HeLa cells for single-molecule imaging: German Collection of Microorganisms and Cell Cultures GmbH (ACC 57); Expi293F cells (ThermoFisher); Expi293F GnTI- (ThermoFisher); IL-17C HEK-Blue NF-kB Reporter cells (Invivogen).
Authentication	HeLa cells (German Collection of Microorganisms and Cell Cultures GmbH), Expi293F cells (ThermoFisher), Expi293F GnTI- (ThermoFisher), and IL-17C HEK-Blue NF-kB Reporter cells (Invivogen) were guaranteed by the suppliers and no additional authentication was performed by the authors of this study.
Mycoplasma contamination	HeLa cells for single-molecule imaging were tested negatively for mycoplasma (PCR). Expi293F cells (ThermoFisher) and Expi293F GnTI- cells (ThermoFisher) used for protein production were not tested for mycoplasma contamination by the authors of this study. The IL-17C HEK-Blue NF-kB Reporter cells (Invivogen) were tested for mycoplasma contamination by the manufacturer and no additional testing was performed by the authors of this study.
Commonly misidentified lines (See ICLAC register)	None of the cell lines used in this study are commonly misidentified.

Human research participants

Policy information about studies involving human research participants

Population characteristics	PBMCs were obtained from the Stanford Blood Center from healthy donors.
Recruitment	The Stanford Blood Center recruited donors. Written informed consent was obtained from the donors prior to tissue collection.
Ethics oversight	The Stanford Blood Center.

Note that full information on the approval of the study protocol must also be provided in the manuscript.
